# Anti‐Allergic Potential of *Chamaecrista nomame* and Its Compound Luteolin for Novel Asthma Therapy

**DOI:** 10.1002/ptr.70363

**Published:** 2026-05-05

**Authors:** Tae Kyeom Kang, Myungsuk Kim, Tam Thi Le, Geon Park, Yuna Jung, Hyobin Jeong, Youngsang Yoo, Wook‐Bin Lee, Sang Hoon Jung

**Affiliations:** ^1^ Center for Natural Product Efficacy Optimization Korea Institute of Science and Technology Gangneung Republic of Korea; ^2^ Department of Natural Product Applied Science University of Science and Technology (UST) Daejeon Republic of Korea; ^3^ Division of Biological Science and Technology Yonsei University Wonju Republic of Korea; ^4^ Department of Systems Biology, College of Life Science and Biotechnology Yonsei University Seoul Republic of Korea; ^5^ Department of Allergy and Clinical Immunology, Gangneung Asan Hospital University of Ulsan College of Medicine Seoul Republic of Korea

**Keywords:** allergic asthma, anti‐allergic effects, *Chamaecrista nomame*, immune modulation, luteolin, mast cell degranulation

## Abstract

Asthma is a chronic inflammatory disease driven by dysregulated immune responses and mast cell activation. While corticosteroids remain the primary treatment, their long‐term use is associated with adverse effects, necessitating safer alternatives. This study aims to evaluate the therapeutic potential and underlying mechanisms of *Chamaecrista nomame* (CN) extract and its active compound luteolin in allergic asthma. We performed bioactivity‐guided fractionation to identify active compounds from CN extract. An ovalbumin‐induced murine asthma model was utilized to investigate the efficacy of CN in vivo. Transcriptomic analysis of bone marrow‐derived mast cells was conducted to elucidate molecular pathways regulated by luteolin. Additionally, cytokine release assays were performed using house dust mite‐stimulated human peripheral blood mononuclear cells (PBMCs). CN extract demonstrated potent anti‐allergic effects by significantly inhibiting mast cell degranulation and inflammatory mediator release. Luteolin was identified as the primary active compound, modulating FcεRI‐mediated signaling in mast cells. In the murine asthma model, CN markedly reduced airway inflammation, mucus hypersecretion, and immune cell infiltration, with efficacy comparable to corticosteroids. Transcriptomic data indicated that luteolin suppresses proinflammatory cytokine production by downregulating NF‐κB and MAPK signaling pathways. Moreover, CN and luteolin significantly inhibited cytokine release from house dust mite‐stimulated human PBMCs, highlighting clinical relevance. These findings suggest that CN and luteolin may serve as promising natural therapeutic agents for allergic asthma, offering a potential alternative to conventional treatments.

## Graphical abstract

*Chamaecrista nomame* (CN) and its active compound luteolin attenuate allergic airway inflammation by inhibiting mast cell activation and suppressing NF‐κB/MAPK signaling, leading to reduced cytokine production and OVA‐specific IgE levels.

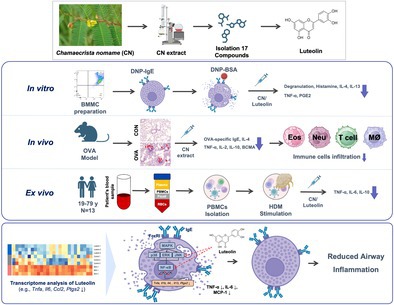

**Abbreviations**
Al(OH)_3_
: aluminum hydroxide
BALF: bronchoalveolar lavage fluid
BMMC: bone marrow‐derived mast cells
CN: *Chamaecrista nomame*
DEX: dexamethasone
H&E: hematoxylin and eosin
OVA: ovalbumin
PAS: periodic acid–Schiff
PBMC: peripheral blood mononuclear cell
PMNs: polymorphonuclear cells
SEAP: secretion of embryonic alkaline phosphatase

## 1 Introduction

Asthma is a chronic inflammatory disorder of the respiratory tract, characterized by debilitating symptoms such as wheezing, coughing, chest tightness, and shortness of breath. It is a major public health concern affecting as many as 260 million people worldwide, with its prevalence on the rise (Zhang et al. 2025). The pathogenesis of asthma involves a complex interplay between genetic and environmental factors, leading to airway inflammation, mucus hypersecretion, and airway remodeling (Lambrecht et al. 2025). Histologically, the disease manifests as thickened submucosa, epithelial cell shedding, and increased infiltration of immune cells such as eosinophils, neutrophils, and T‐cells in the peribronchial area. This heightened immune cell infiltration exacerbates airway and lung inflammation, intensifies airway hyperresponsiveness, and increases asthma severity.

Among the various cell types involved in asthma, mast cells play a critical role in initiating and perpetuating the inflammatory response (Xie et al. 2024). These cells, which are distributed throughout the body, including the respiratory tract, are readily activated by various triggers, including repeated allergen exposure (Xie et al. 2024). Upon activation, mast cells undergo IgE‐mediated aggregation of FcεRI on their surface, leading to the rapid release of inflammatory mediators through degranulation. Following FcεRI aggregation, mast cells release a wide array of preformed and newly synthesized mediators, including histamine, leukotrienes, prostaglandins, and cytokines. These mediators contribute to bronchoconstriction, increased mucus secretion, and inflammation, which collectively lead to the characteristic symptoms of allergy and asthma.

Current asthma treatments primarily rely on bronchodilators and inhaled corticosteroids, aimed at improving airflow and reducing inflammation, respectively (Çelik et al. 2023). However, the long‐term use of corticosteroids can lead to adverse effects such as growth suppression, osteoporosis, and adrenal suppression (Ottesen et al. 2025), and many patients continue to struggle with inadequate symptom control. Moreover, a significant proportion of patients with severe asthma remain unresponsive to conventional treatments, emphasizing the need for alternative therapies with better safety and efficacy (Sin and Busse 2024).

In this context, natural products, particularly those from medicinal plants with a history of traditional use, are gaining attention as potential anti‐asthmatic agents. *Chamaecrista nomame* (CN) (Siebold) H. Ohashi, a plant in the Fabaceae family, has been traditionally used in Asian medicine for its anti‐obesity, antioxidant, and anti‐inflammatory properties, attributed to phytochemicals like quercitrin, protocatechuic acid, and ferulic acid (Zibaee et al. 2023). However, its specific immunomodulatory effects, particularly in regulating mast cell‐mediated allergic responses in asthma, remain largely unexplored.

Our study aimed to identify novel plant‐derived compounds with anti‐allergic properties for asthma treatment. Through a comprehensive screening of 302 plant extracts, we identified the ethanol extract of CN as a promising candidate due to its potent anti‐degranulation activity. In an ovalbumin (OVA)‐induced mouse model of allergic asthma, CN significantly inhibited allergic reactions. We also explored the mechanistic role of luteolin, a key compound in CN, in mast cells, demonstrating its ability to modulate critical signaling pathways involved in allergic responses. Finally, we validated the anti‐allergic effects of CN and luteolin in human PBMCs stimulated with HDM extract, a clinically relevant allergen. These results highlight the therapeutic potential of CN and luteolin as novel, natural‐derived agents for treating allergic asthma.

## 2 Materials and Methods

### 2.1 Plant Material and Extraction

Dried CN leaves were purchased from a commercial market (Samhong, Gyeonggi‐do, Korea). The dried leaves (4 kg) were extracted with 70% ethanol (24 L) for 3 days at room temperature. The supernatant was filtered using Whatman No. 1 filter paper (Whatman, Buckinghamshire, UK), and the extraction process was repeated three additional times with the remaining powder. The combined filtrates were evaporated under vacuum at 45°C using a Rotavapor R‐100 (Büchi, Flawil, Switzerland), yielding 390 g of crude extract. The ethanol extract of CN was stored at −20°C until further use in experiments.

### 2.2 Isolation and Identification

The CN was suspended in water and partitioned sequentially with *n*‐hexane (Hx) and ethyl acetate (EA) to obtain the Hx fraction (10 g), EA fraction (100 g), and a residual aqueous layer (Figure S1). Further details on the isolation and identification of CN can be found in Appendix S11.

## 3 Results

### 3.1 CN Extract Potently Inhibits Mast Cell Degranulation and Inflammatory Mediator Release In Vitro

**FIGURE 1:**
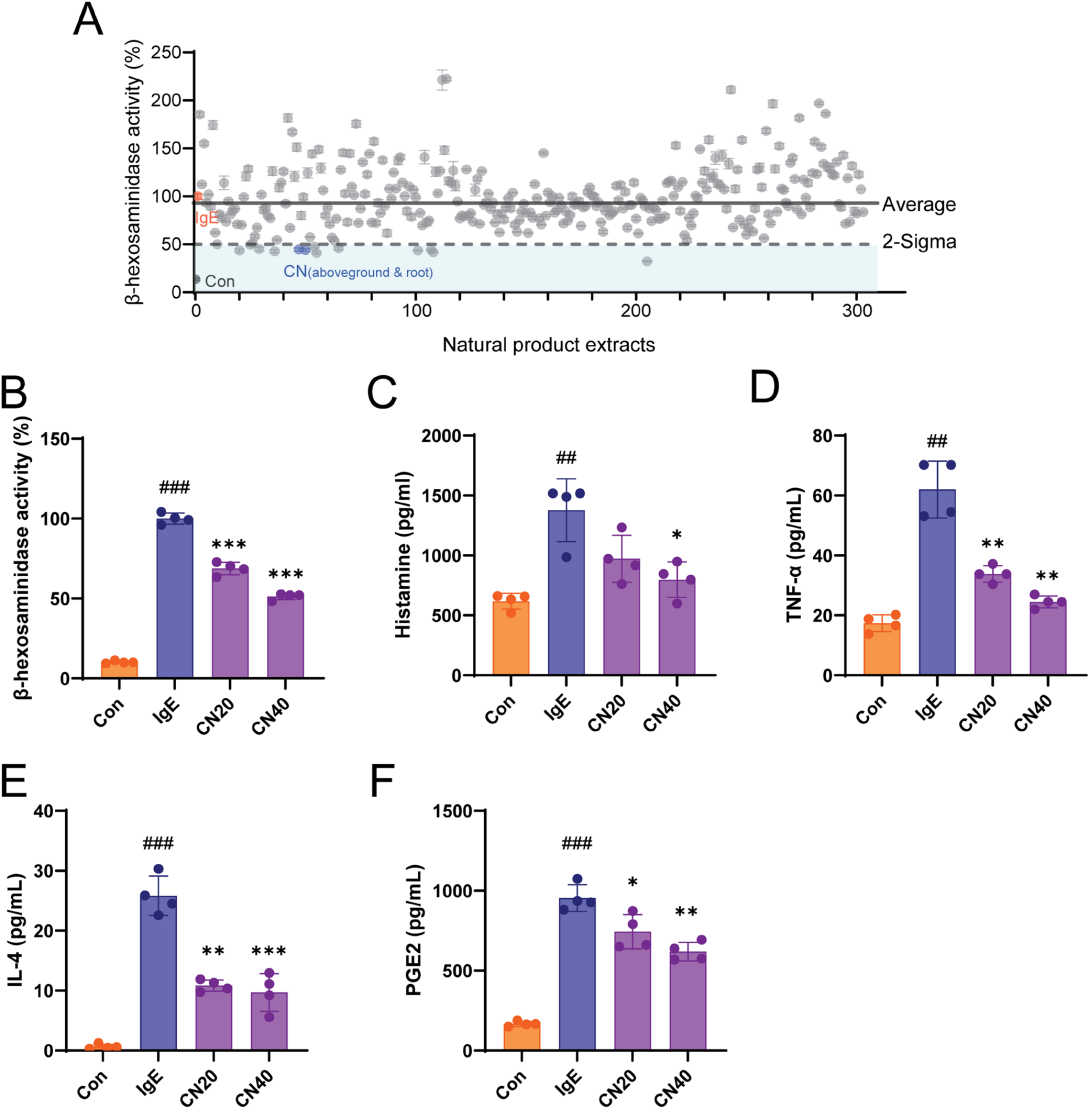
*Chamaecrista nomame* (CN) extract inhibits IgE‐mediated degranulation and inflammatory mediator release in RBL‐2H3 mast cells. (A) Screening of 302 natural product extracts for their ability to inhibit β‐hexosaminidase release in IgE‐sensitized, antigen‐stimulated RBL‐2H3 cells. Each dot represents a single extract tested at a fixed concentration. The red line indicates the average β‐hexosaminidase activity, while the blue dashed line represents the 2‐sigma threshold. The CN extract from both aboveground and root parts is highlighted as a significant inhibitor. (B–F) Dose‐dependent effects of CN on mediator release in RBL‐2H3 cells. Cells were sensitized with anti‐DNP‐IgE and then treated with CN at concentrations of 20 μg/mL (CN20) or 40 μg/mL (CN40) for 1 h prior to challenge with DNP‐BSA. (B) β‐hexosaminidase activity was measured after stimulation with DNP‐BSA. (C) Histamine levels were quantified using an ELISA. (C–E) The levels of proinflammatory cytokines TNF‐α (D), IL‐4 (E), and PGE2 (F) were also measured using ELISA. Data are expressed as the mean ± SEM. *p < 0.05, **p < 0.01, ***p < 0.001 versus the vehicle group; ^##^ *p* < 0.01, ^###^ *p* < 0.001 versus the control group.

To identify potential plant extracts for the treatment of allergic asthma, we conducted a comprehensive in vitro screening of 302 plant extracts to evaluate their capacity to suppress degranulation. Ethanol extracts derived from these 302 indigenous plant species in Korea were tested for their ability to inhibit β‐hexosaminidase release, a marker for degranulation, in the RBL‐2H3 basophil cell line following treatment with DNP‐IgE/DNP‐BSA. Thirteen extracts exhibited significant suppression, exceeding the 50% threshold (defined as two standard deviations below the mean) of β‐hexosaminidase activity. Notably, CN showed inhibitory effects in both the aboveground and root extracts (Figure 1A). Based on these observations, we proceeded with an in‐depth investigation of CN.

No significant cytotoxic effects were observed in RBL‐2H3 cells treated with CN, even at doses up to 100 μg/mL (Figure S2A). We then evaluated the anti‐degranulation activity of the total ethanol extract, along with hexane and EA fractions of CN. All three fractions significantly inhibited β‐hexosaminidase release (Figure S2B), indicating that multiple active compounds may contribute to CN's anti‐allergic effects. Additionally, tests were performed on ethanol extracts of CN at varying concentrations, ranging from 0% to 95%. The results showed that extracts containing ethanol concentrations of 50%, 70%, and 95% effectively inhibited degranulation (Figure S2C).

To further assess the anti‐allergic properties of CN, we examined its effects on the release of key inflammatory mediators implicated in asthma pathogenesis. The RBL‐2H3 cells, which model mast cell and basophil activity, were stimulated with DNP‐IgE/DNP‐BSA to induce degranulation and cytokine release. Elevated levels of histamine, TNF‐α, IL‐4, and PGE2 were observed following DNP‐IgE/DNP‐BSA treatment. However, CN significantly inhibited the release of these mediators in a dose‐dependent manner (Figure 1B–F). Collectively, these findings demonstrate that CN extract potently inhibits mast cell degranulation and the release of multiple inflammatory mediators in vitro, supporting its potential as a therapeutic agent for allergic asthma.

### 3.2 CN Alleviates Airway Inflammation and Modulates Immune Cell Infiltration in an OVA‐Induced Asthma Model

**FIGURE 2:**
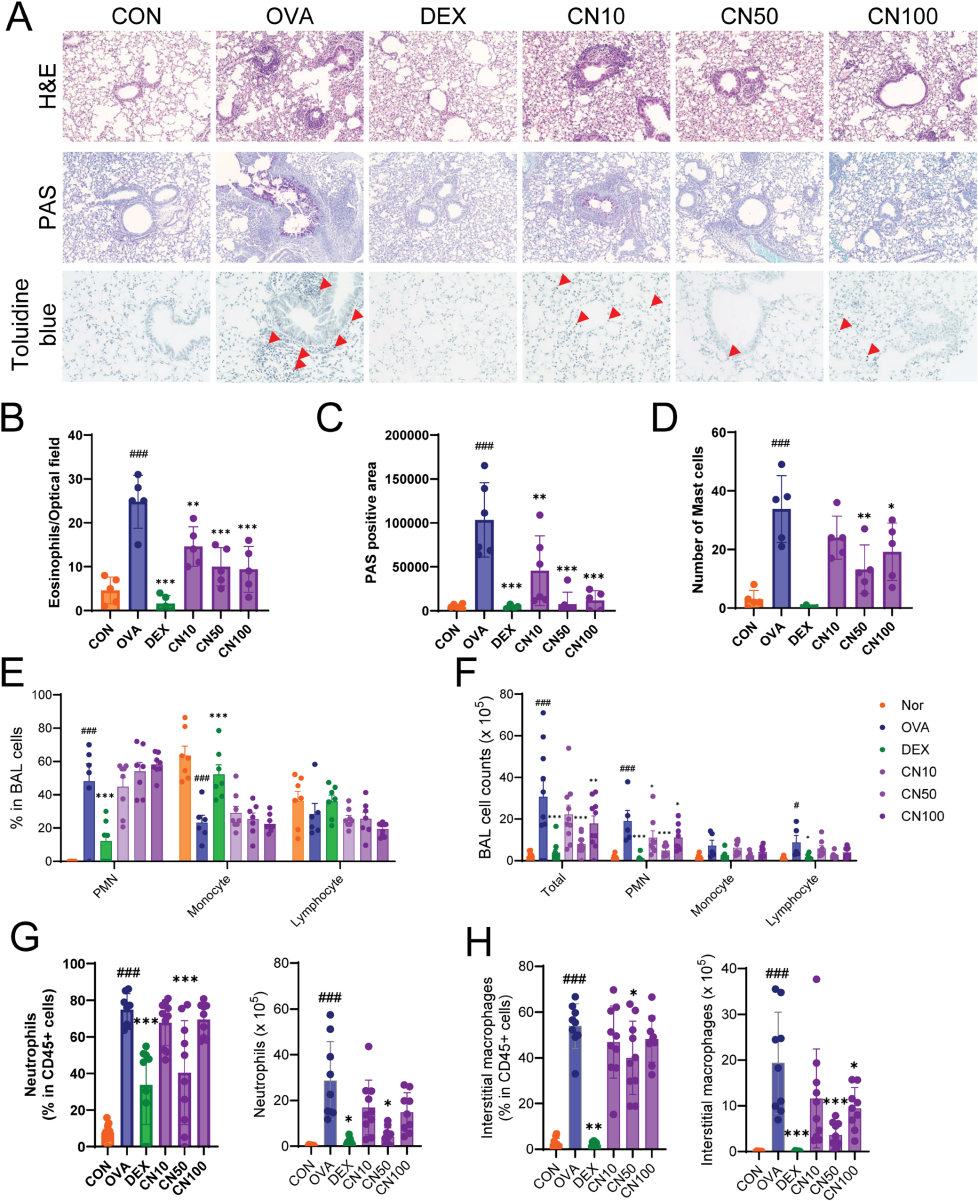
Histological analysis of lung tissue and BALF immune cell infiltration following CN treatment in an OVA‐induced asthma model. (A) Representative images of lung sections from each treatment group stained with H&E (top row), PAS (middle row), and Toluidine blue (bottom row) at ×200 magnification. The groups include Control (CON), OVA‐challenged mice (OVA), OVA‐challenged mice treated with dexamethasone (DEX, 2.5 mg/kg), and OVA‐challenged mice treated with CN (CN10, CN50, CN100; 10, 50, and 100 mg/kg, respectively). (B) Quantification of eosinophils per optical field from H&E‐stained sections. (C) Quantification of the PAS‐positive area. (D) Quantification of mast cell infiltration in lung tissue. (E, F) The percentage (E) and absolute number (F) of immune cells in BALF were analyzed. The percentage and absolute number of neutrophils (G, CD45 + CD11b + Ly‐6G+), interstitial macrophages (H, CD45 + CD11c‐F4/80+) in BALF were evaluated. Data are expressed as the mean ± SEM (*n* = 6 per group). Significant differences were observed at ^###^ *p* < 0.001 versus the Control group; **p < 0.01, ***p < 0.001 versus the OVA group.

To validate the in vitro anti‐allergic properties of CN, we assessed its efficacy in a mouse model of OVA‐induced asthma. Sensitization and OVA challenges were conducted over a 28‐day period, during which CN was orally administered for 14 days at varying doses (10, 50, and 100 mg/kg), with dexamethasone (DEX) serving as a positive control at 2.5 mg/kg (Figure S3). Histopathological evaluation of lung tissues using H&E staining revealed severe peribronchiolar and perivascular infiltration of inflammatory cells in the OVA group. Additionally, PAS staining indicated excessive mucus production in the OVA group. However, treatment with CN significantly reduced inflammatory cell infiltration and markedly inhibited mucus hypersecretion, with effects comparable to those observed with DEX. Furthermore, toluidine blue staining revealed a marked increase in mast cell numbers in the OVA‐challenged group, whereas CN treatment significantly reduced mast cell infiltration and degranulation within the airway tissues. These findings provide direct in vivo evidence that CN alleviates allergic airway inflammation, at least in part, through the inhibition of mast cell activation. (Figure 2A–D).

To further investigate the effects of CN on allergic inflammation, we analyzed the cellular composition of bronchoalveolar lavage fluid (BALF). Comparative analysis revealed a significant elevation in the numbers of total inflammatory cells, polymorphonuclear cells (PMNs), and lymphocytes in the BALF of the OVA group compared to the control group. However, administration of CN led to a notable reduction in the number of inflammatory cells in BALF, particularly PMNs, compared to the OVA group (Figure 2E,F). Further analysis focused on distinct inflammatory cell types in BALF. Notably, treatment with 50 mg/kg CN resulted in a reduction in both the percentage and number of neutrophils compared to the OVA group (Figure 2G). Interestingly, CN also decreased the numbers of alveolar and interstitial macrophages (Figure 2H, Figure S4A), while having no significant impact on dendritic cells (DC) or B cells (Figure S4B,C). Additionally, CN significantly reduced the number of T cells in BALF (Figure S4D). These results suggest that CN has a broad ability to mitigate inflammatory cell infiltration in asthmatic mice, with significant effects on neutrophils, alveolar and interstitial macrophages, and T cells.

**FIGURE 3:**
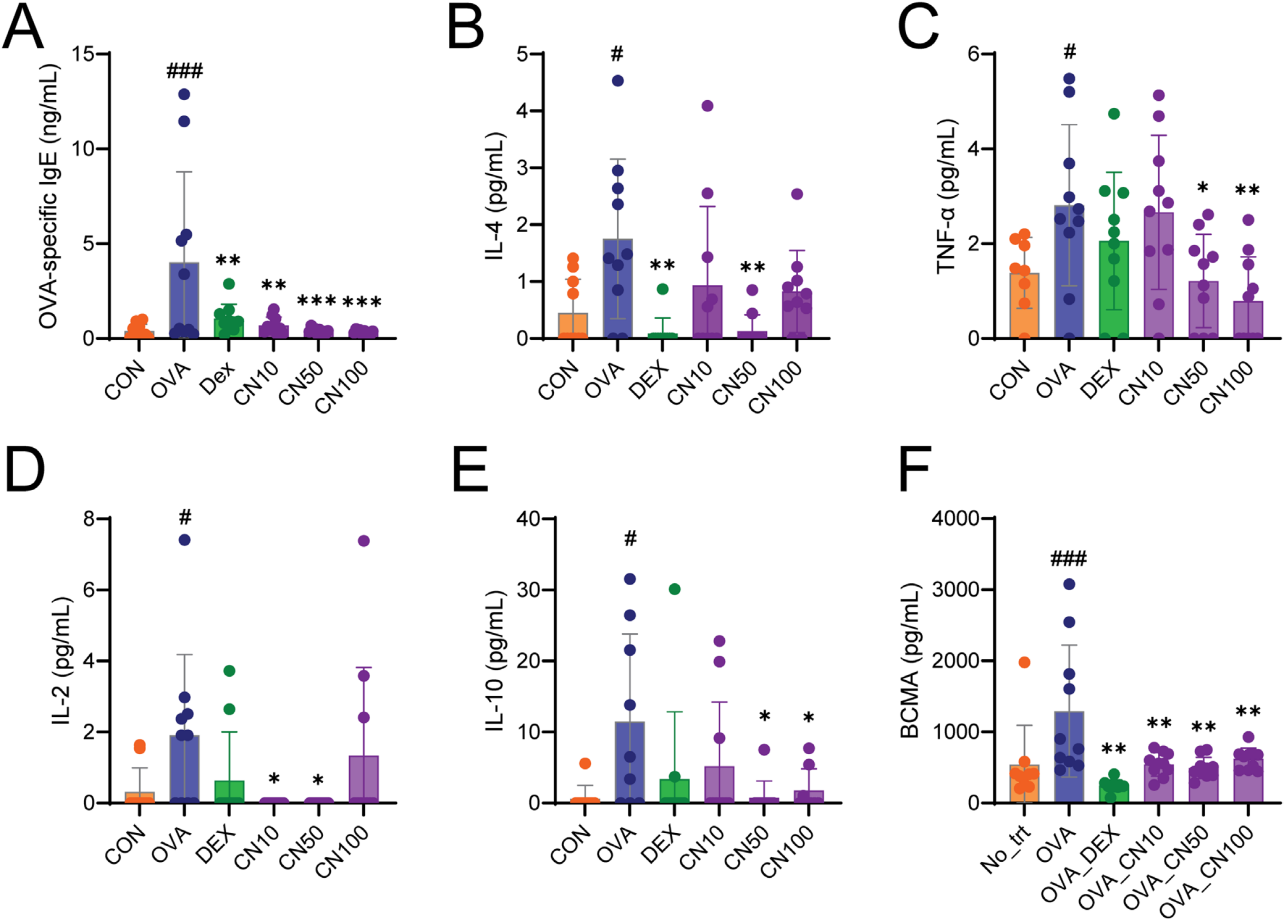
Decreased levels of inflammatory cytokines and OVA‐specific immunoglobulin in asthmatic mice following CN treatment. Serum levels of (A) OVA‐specific IgE, (B) IL‐4, (C) TNF‐α, (D) IL‐2, (E) IL‐10, and (F) BCMA were measured using ELISA in the different treatment groups. Data are presented as mean ± SEM. Significant differences at ^#^ *p* < 0.05, ^###^ *p* < 0.001 versus the Control group; *p < 0.05, **p < 0.01, ***p < 0.001 versus the OVA group.

In the context of allergic asthma, the activation of Th2 cells is closely associated with inflammatory reactions. Th2‐related cytokines play a pivotal role in eosinophil activation and the induction of IgE production by B cells (Ogulur et al. 2025). A comparative study revealed a significant increase in the production of OVA‐specific IgE, as well as IL‐4, TNF‐α, IL‐2, IL‐10, and BCMA, following OVA sensitization and challenge compared to the control group (Figure 3A–F). However, the administration of CN effectively suppressed the increase in these cytokines, showing results comparable to those observed in the DEX treatment group. These findings suggest that CN may ameliorate allergic airway inflammation by modulating Th2 cell responses and reducing OVA‐specific IgE production.

### 3.3 Bioactivity‐Guided Fractionation of CN Identifies Luteolin as a Potent Inhibitor of Mast Cell Activation

**FIGURE 4:**
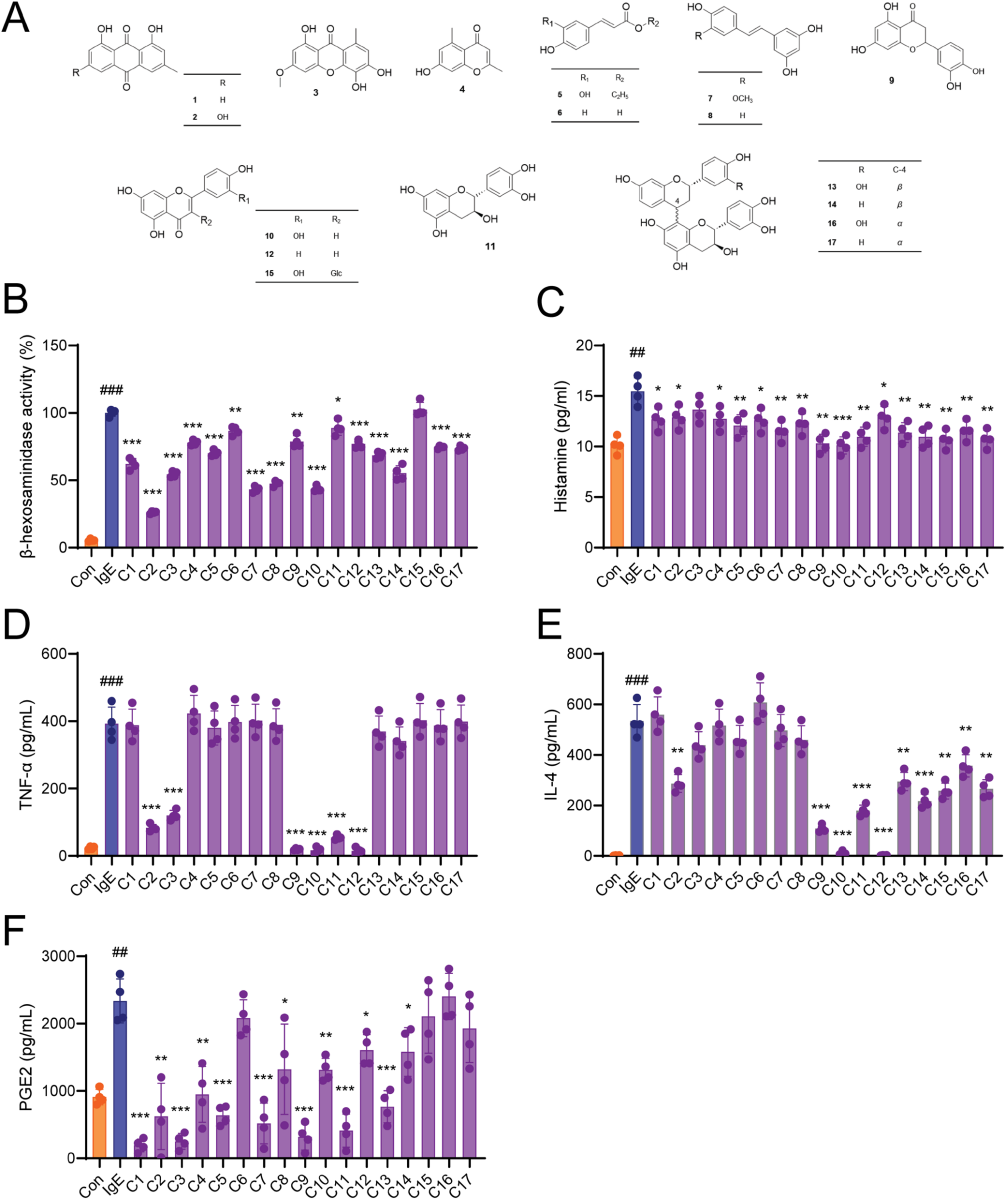
HPLC‐DAD analysis of CN extract and the effects of isolated compounds on IgE‐mediated activation of RBL‐2H3 mast cells. (A) The numbered peaks correspond to specific compounds isolated from CN Chemical structures of the 17 compounds isolated from CN. (B–F) Effects of isolated compounds (1–17) on IgE‐mediated responses in RBL‐2H3 mast cells. The cells were sensitized with anti‐DNP‐IgE and then treated with the 17 isolated compounds (40 μM) for 1 h prior to challenge with DNP‐BSA. The following parameters were measured: (B) β‐hexosaminidase release, (C) histamine release, (D) TNF‐α production, (E) IL‐4 production, and (F) PGE2 production. C1, chrysophanol; C2, emodin; C3, drimiopsin H; C4, altechromone A; C5, ethyl caffeate; C6, p‐coumaric acid; C7, isorhapontigenin; C8, resveratrol; C9, eriodictyol; C10, luteolin; C11, catechin; C12, apigenin; C13 and C16, (2S)‐3′,4′,7‐trihydroxyflavan‐(4β⟶8)‐catechin/(2S)‐3′,4′,7‐trihydroxyflavan‐(4α⟶8)‐catechin; C14, (2S)‐4′,7‐dihydroxyflavan‐(4β⟶8)‐catechin; C15, isoquercitrin; and C17, (2S)‐4′,7‐dihydroxyflavan‐(4α⟶8)‐catechin. Data are expressed as the mean ± SEM. ^##^ *p* < 0.01, ^###^ *p* < 0.001 versus the control group; *p < 0.05, **p < 0.01, ***p < 0.001 versus the IgE group.

To identify the active constituents responsible for the observed anti‐allergic effects of CN, we employed a bioactivity‐guided fractionation approach. The crude EA fraction of CN underwent column chromatography, which led to the isolation and identification of 17 compounds: chrysophanol (**1**) (Mari et al. 2019), emodin (**2**) (Mari et al. 2019), drimiopsin H (**3**) (Zhuang et al. 2015), altechromone A (**4**) (Kashiwada et al. 1984), ethyl caffeate (**5**) (Xiang et al. 2011), p‐coumaric acid (**6**) (Xiang et al. 2011), isorhapontigenin (**7**) (Fernández‐Marín et al. 2012), resveratrol (**8**) (Commodari et al. 2005), eriodictyol (**9**) (Chu et al. 2016), luteolin (**10**) (Lin et al. 2015), catechin (**11**) (Davis et al. 1996), apigenin (**12**) (Darmawan et al. 2015), (2S)‐3′,4′,7‐trihydroxyflavan‐(4β⟶8)‐catechin (**13**) (Hatano et al. 1997), (2S)‐4′,7‐dihydroxyflavan‐(4β⟶8)‐catechin (**14**) (Hatano et al. 1997), isoquercitrin (**15**), (2S)‐3′,4′,7‐trihydroxyflavan‐(4α⟶8)‐catechin (**16**) (Hatano et al. 1997), and (2S)‐4′,7‐dihydroxyflavan‐(4α⟶8)‐catechin (**17**) (Hatano et al. 1997). HPLC with diode‐array detection identified seven compounds at retention times of 15.72 (compound **11**), 24.43 (compound **15**), 25.48 (compounds **13** and **16**), 36.52 (compound **10**), 43.36 (compound **2**), and 45.06 (compound **1**) min, at 280 nm (Figure 4A, Figure S5). These identifications were based on comparisons of the retention times and absorption spectra of the unknown peaks in the extract with data from the isolated compounds.

Next, we assessed the contribution of each isolated compound to the overall anti‐allergic activity of CN by evaluating their ability to inhibit IgE‐mediated mast cell activation in RBL‐2H3 cells. We measured degranulation, histamine release, and the production of TNF‐α, IL‐4, and PGE2. Notably, compounds C2 (emodin), C9 (eriodictyol), C10 (luteolin), C11 (catechin), and C12 (apigenin) exhibited significant inhibitory effects across all five assays, with luteolin demonstrating the most remarkable efficacy (Figure 4B–F). Given its remarkable efficacy, luteolin was selected for further investigation to elucidate the mechanisms underlying its inhibitory effects on allergic asthma.

### 3.4 Luteolin Inhibits Degranulation andSuppresses Th2 Cytokine Production in Bone Marrow‐Derived Mast Cells

To determine the regulatory effect of luteolin on mast cell activation, we thoroughly examined its influence on granule release and the production of Th2‐related cytokines in mouse mast cells, using a widely recognized in vitro differentiation method from bone marrow (Bawazir et al. 2024). We developed BMMCs with IL‐3‐enriched medium for a minimum of 4 weeks, resulting in a high‐purity c‐kit^+^FcεRI^+^ population (Figure S6). When exposed to an IgE‐antigen complex, luteolin effectively prevented the release of β‐hexosaminidase, indicating its ability to suppress mast cell degranulation (Figure S7A). Given the critical role of Th2 cytokines, such as IL‐4 and IL‐13, in driving allergic inflammation and their established link to mast cell activation, we next examined the effect of luteolin on cytokine production. Our results showed that luteolin significantly reduced the release of IL‐4 and IL‐13 by BMMCs stimulated with DNP‐IgE/DNP‐BSA (Figure S7B,C), demonstrating its ability to suppress allergic reactions mediated by mast cells. These results provide mechanistic insight into the anti‐allergic properties of luteolin and suggest that its ability to target mast cell activation contributes to the therapeutic effects of CN extract observed in vivo.

### 3.5 Luteolin Modulates the BMMCs Transcriptome, Suppressing Key Inflammatory Signaling Pathways

**FIGURE 5:**
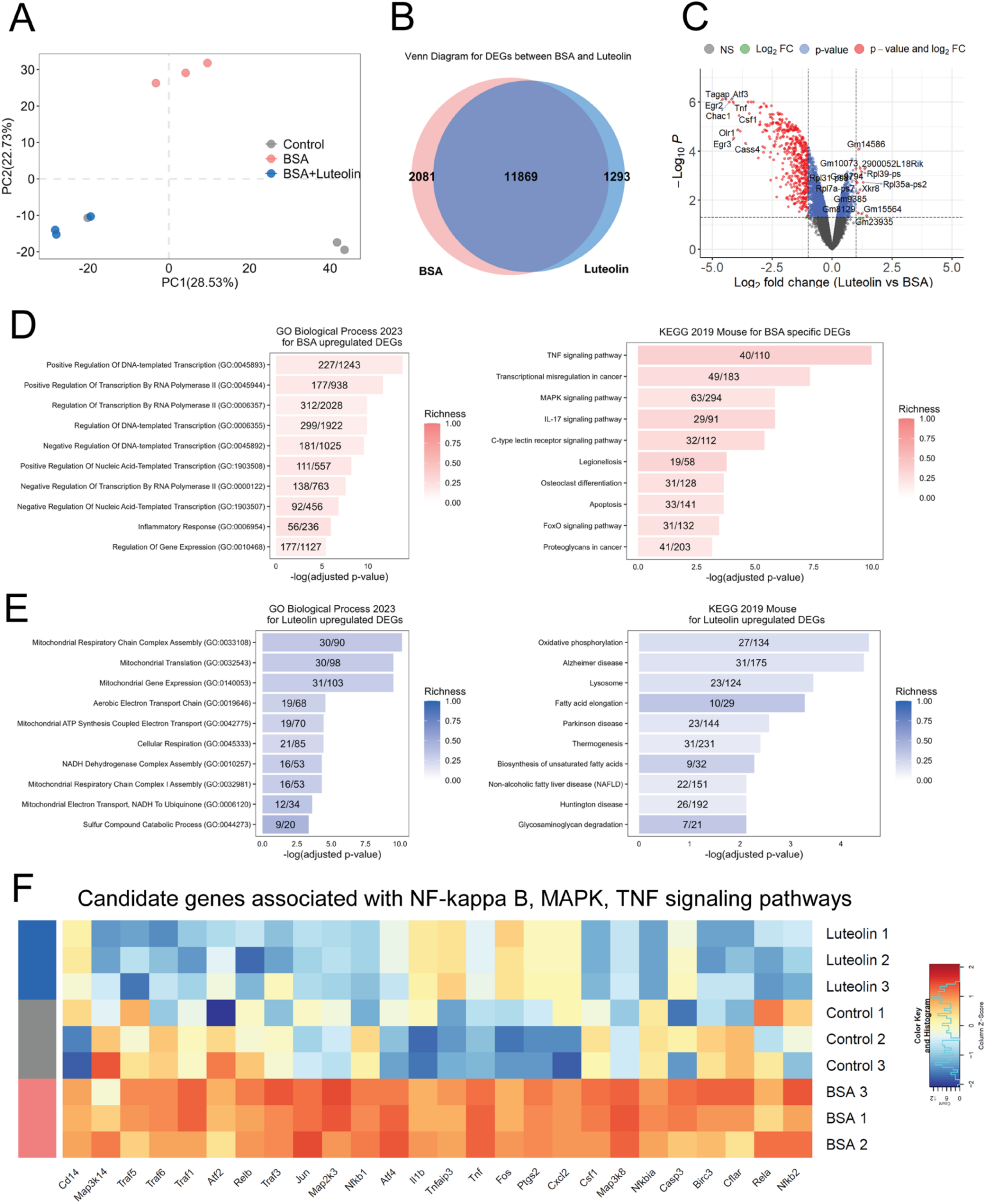
Transcriptome analysis identifying the regulatory effects of luteolin from CN on intracellular signaling pathways in BMMCs. (A) Principal component analysis (PCA) of RNA sequencing data from bone marrow‐derived mast cells (BMMCs). Each dot represents a single sample, colored by treatment group: Control (untreated), BSA (DNP‐BSA stimulated), and BSA + luteolin (DNP‐BSA stimulated with luteolin pretreatment). The PCA plot demonstrates distinct clustering of samples based on treatment, indicating significant alterations in gene expression. (B) Venn diagram illustrating the number of differentially expressed genes (DEGs) in BMMCs following stimulation with DNP‐BSA (BSA) and in BMMCs treated with luteolin after DNP‐BSA stimulation (luteolin). DEGs were identified using an adjusted *p* < 0.05. (C) Volcano plot depicting the differential gene expression between DNP‐BSA‐stimulated BMMCs treated with luteolin and those treated with vehicle. The *x*‐axis represents the log_2_ (fold change) in gene expression (luteolin/BSA), and the y‐axis represents the −log_10_ (adjusted *p* value). Each dot represents a gene. Significantly upregulated genes are shown in red, and significantly downregulated genes are shown in blue. Key genes mentioned in the text are labeled. (D, E) Gene Ontology (GO) and KEGG pathway enrichment analyses for upregulated and downregulated DEGs in luteolin‐treated BMMCs. The top enriched GO terms (biological process) and KEGG pathways are shown, ranked by adjusted *p* value. The bar graphs depict the number of genes associated with each term (gene count) and the richness factor (ratio of observed to expected number of DEGs within each term). (F) Heatmap illustrating the normalized expression levels of selected genes involved in NF‐κB, MAPK, and TNF signaling pathways. Each row represents a gene, and each column represents a sample. The color scale indicates the relative expression level of each gene (red: higher expression; blue: lower expression).

Mast cells are predominantly tissue‐resident granulocytes that play a key role in allergic responses (Wang et al. 2024). To further delineate the molecular mechanisms underlying these effects on BMMCs, we performed RNA sequencing on cells stimulated with DNP‐IgE/DNP‐BSA, both with and without luteolin treatment. Principal component analysis (PCA) revealed distinct clusters based on treatment (Figure 5A), indicating significant transcriptome alterations. Differential gene expression analysis identified 3374 differentially expressed genes (DEGs) following luteolin treatment, with a notable downregulation of 2081 genes, suggesting that a primary effect of luteolin is the suppression of gene expression (Figure 5B). A volcano plot further highlighted the genes most significantly affected by luteolin treatment, with many downregulated genes associated with inflammatory processes (Figure 5C). Subsequent analysis using Gene Ontology (GO) and KEGG pathway enrichment revealed that luteolin significantly modulated several biological processes, with a prominent enrichment of downregulated genes involved in key inflammatory signaling pathways such as NF‐kB, MAPK, and TNF signaling (Figure 5D,E). A heatmap of DEG expression levels further supports this finding, showing a distinct downregulation of genes within these pathways (Figure 5F). Importantly, cell viability assays confirmed that the concentrations of luteolin used in these experiments did not induce cytotoxicity in BMMCs, indicating that the observed transcriptomic changes reflect regulatory effects rather than secondary responses to cell stress (Figure S8). These findings demonstrate that luteolin profoundly alters the transcriptome of activated mast cells, specifically suppressing the expression of genes associated with critical inflammatory signaling pathways, including NF‐κB, MAPK, and TNF pathways.

### 3.6 Luteolin Suppresses NF‐kB and MAPK Signaling Pathways in BMMCs

**FIGURE 6:**
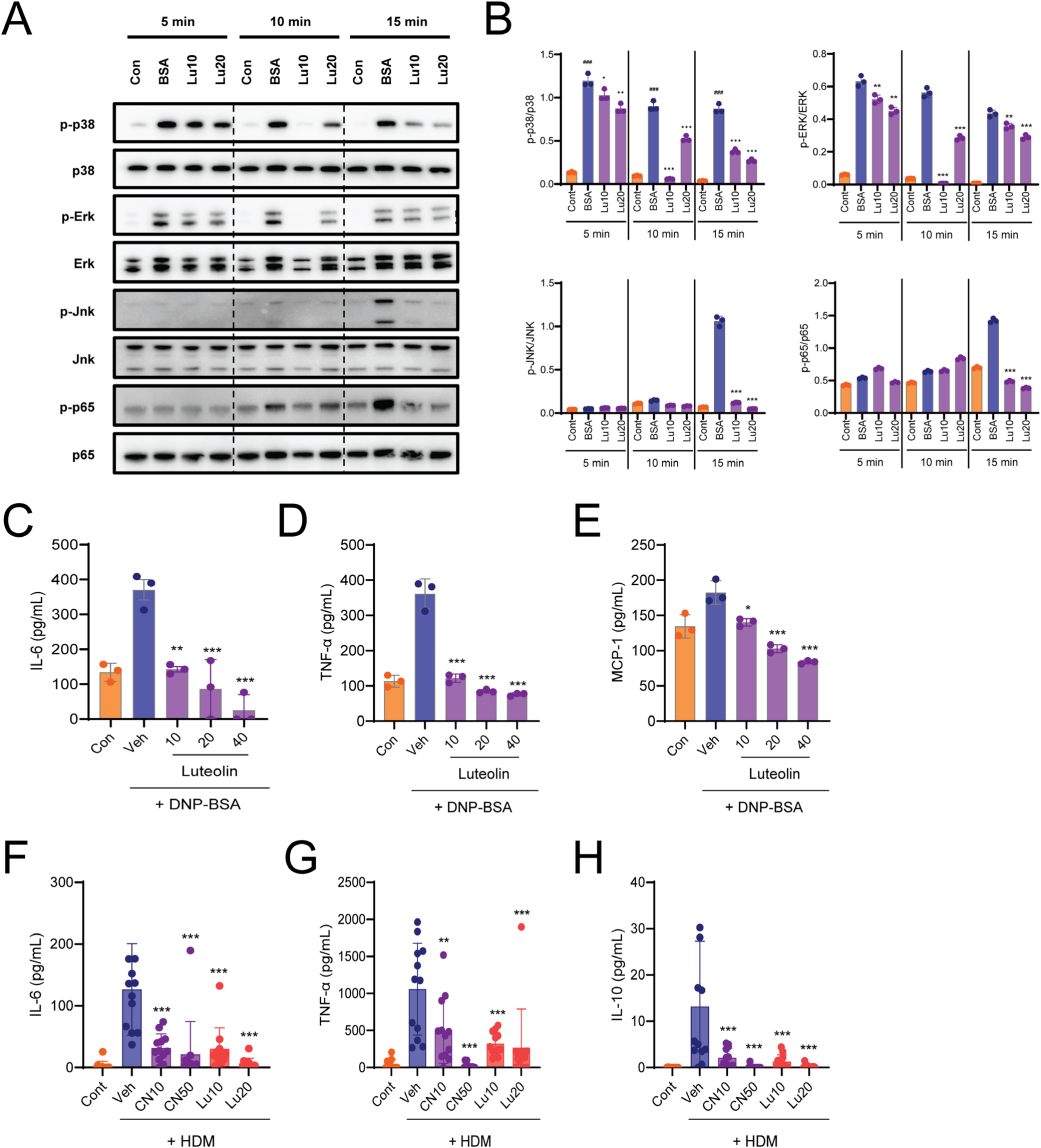
Luteolin inhibits NF‐κB and MAPK signaling pathways in activated BMMCs and suppresses cytokine production in HDM‐stimulated human PBMCs. (A, B) BMMCs were sensitized with anti‐DNP‐IgE and then treated with luteolin for 1 h prior to challenge with DNP‐BSA. (A) Immunoblot analysis showing the phosphorylation levels of p65, p38, Erk, and Jnk at different time points (5, 10, and 15 min). (B) Quantification of the relative band intensities for phospho‐p65, phospho‐p38, phospho‐Erk, and phospho‐Jnk, normalized to total protein levels. (C–E) Effects of luteolin on proinflammatory cytokine and chemokine production in activated BMMCs. Cells were sensitized and pretreated as in (A) and stimulated with DNP‐BSA for 6 h (or other time as appropriate). The levels of (C) IL‐6, (D) TNF‐α, and (E) MCP‐1 in the cell culture supernatants were measured by ELISA. (F–H) Effects of CN extract and luteolin on cytokine production in HDM‐stimulated human PBMCs. PBMCs were isolated from human donors (ages 19–79, *n* = 13) and stimulated with house dust mite (HDM) in the presence or absence of CN extract (10 or 50 μg/mL) or luteolin (10 or 20 μM) for 24 h. The levels of (F) IL‐6, (G) TNF‐α, and (H) IL‐10 in the cell culture supernatants were measured by multiplex ELISA. Data are presented as mean ± SEM. Significant differences at ^###^ *p* < 0.001 versus the Control group; *p < 0.05, **p < 0.01, ***p < 0.001 versus the Vehicle group.

To validate the findings of our transcriptomic analysis and further elucidate the molecular mechanisms underlying the anti‐allergic effects of luteolin, we examined its impact on key signaling pathways involved in mast cell activation. Immunoblot analysis of lysates from DNP‐IgE/DNP‐BSA‐stimulated BMMCs revealed that luteolin significantly reduced the phosphorylation of p65 (NF‐κB), p38 (MAPK), ERK (MAPK), and JNK (MAPK), indicating inhibition of these signaling pathways (Figure 6A,B). To further support our findings, we assessed the effect of luteolin on key cytokine production associated with NF‐κB and MAPK signaling pathways. qRT‐PCR analysis revealed that luteolin treatment significantly downregulated the expression of Tnf‐α, IL‐1β, and Ptgs2 (COX‐2) in activated BMMCs (Figure S7D–F). Additionally, ELISA assays demonstrated that luteolin significantly suppressed the secretion of IL‐6, TNF‐α, and MCP‐1 in DNP‐BSA‐stimulated BMMCs (Figure 6C–E). Collectively, these results provide strong evidence that luteolin inhibits mast cell activation, at least in part, by suppressing the NF‐κB and MAPK signaling pathways, leading to decreased expression and secretion of proinflammatory cytokines.

### 3.7 CN and Luteolin Suppress Inflammatory Cytokine Production in Human PBMCs Stimulated With HDM Extract

To further validate the therapeutic potential of CN and luteolin in a clinically relevant context, we investigated their effects on cytokine production in human PBMCs stimulated with HDM extract, a common asthma allergen. PBMCs isolated from human donors (ages 19–79, *n* = 13) were stimulated with HDM in the presence or absence of CN or luteolin. HDM stimulation significantly increased the production of proinflammatory cytokines IL‐6, TNF‐α, and notably, IL‐10, compared to unstimulated cells. Both CN and luteolin significantly suppressed HDM‐induced production of IL‐6, TNF‐α, and IL‐10 in a dose‐dependent manner (Figure 6F–H). The significant inhibition of key inflammatory cytokines, particularly IL‐6 and TNF‐α, supports their therapeutic potential in allergic airway diseases. Additionally, the decrease in IL‐10, a cytokine typically known to dampen inflammation, suggests that CN and luteolin modulate the overall inflammatory response. These findings suggest the ability of CN and luteolin to effectively modulate the inflammatory response in human immune cells exposed to a clinically relevant allergen.

## 4 Discussion

This study shows that CN and its active compound luteolin reduce allergic inflammation in both in vitro mast cell models and an OVA‐induced asthma model. CN administration decreased airway inflammation and tissue remodeling, while both CN and luteolin suppressed mast cell degranulation and proinflammatory cytokine release. These effects were associated with regulation of NF‐κB and MAPK signaling, indicating that their activity spans multiple stages of the allergic response rather than acting at a single immunological point.

At the cellular level, in vitro assays demonstrated that CN reduces early mast cell activation, including the release of histamine, TNF‐α, IL‐4, and PGE2. This discovery of CN's anti‐allergic potential represents a novel finding to our knowledge. Further isolation of CN's active components yielded 17 compounds, among which luteolin showed the most pronounced inhibitory activity on mast cell degranulation and cytokine release, consistent with its known anti‐inflammatory mechanisms (Quan et al. 2024; Tsilioni and Theoharides 2024). However, CN as a whole extract exerted comparable effects through multi‐component synergy, rather than dependence on a single active molecule. Other CN‐derived constituents, including emodin, resveratrol, and eriodictyol, also reduced mast cell activation and cytokine production (Werner et al. 2024), supporting a synergistic, multi‐component regulatory mechanism that may enhance reproducibility and robustness in complex immune environments.

In the OVA‐induced asthma model, CN administration reduced inflammatory cell infiltration in BALF and lung tissue and lowered Th2‐related cytokines and IgE levels (Eggel et al. 2024). Notably, CN administered at 50 mg/kg produced stronger anti‐allergic effects than at 100 mg/kg, indicating a nonlinear, bell‐shaped dose–response pattern. Similar phenomena are frequently reported for botanical extracts and immunomodulators, where multi‐component interactions, receptor saturation, or feedback mechanisms lead to maximal efficacy at intermediate doses (Bisson et al. 2015). These results suggest that CN and luteolin operate within an optimal therapeutic window, and further pharmacokinetic and dose–response analyses will be needed to define this range more precisely.

Transcriptome analysis of luteolin‐treated BMMCs showed modulation of NF‐κB and MAPK signaling pathways, as well as genes related to oxidative and adhesion processes (Dileepan et al. 2023), indicating that luteolin affects multiple regulatory nodes in mast cell activation. Consistent effects were observed in HDM‐stimulated PBMCs, where CN and luteolin reduced TNF‐α, IL‐6, and IL‐10 production, suggesting broader cytokine network modulation beyond mast cells. Although RNA‐seq also indicated changes in cell‐cycle and mitochondrial gene programs, cell‐viability assays confirmed that the concentrations used did not induce cytotoxicity in BMMCs. Meanwhile, high concentrations of luteolin have been reported to reduce macrophage viability (Cicia et al. 2025), suggesting that its effects are dependent on both dose and cell type.

While this study demonstrates the anti‐allergic effects of CN and luteolin, further research is needed to fully explore their therapeutic potential. One limitation of this study is the absence of AHR measurement, which should be addressed in future experiments to functionally validate airway improvement. While the OVA‐induced asthma mouse model is widely used, incorporating additional models that better represent the complexity of human asthma, including various phenotypes, would provide more relevant insights. Pharmacokinetic and bioavailability analyses will also be crucial for determining effective dosing ranges and understanding the systemic behavior of CN constituents. Collectively, these future directions will help bridge the translational gap between preclinical findings and clinical application.

In conclusion, CN and luteolin attenuate allergic inflammation by suppressing mast cell activation and modulating key inflammatory signaling pathways. Their multi‐component and multi‐level regulatory actions suggest potential value as complementary interventions for allergic airway disease. Further in vivo, mechanistic, and translational studies will be required to define therapeutic parameters and clinical applicability.

## Author Contributions

**Tae Kyeom Kang:** conceptualization, methodology, investigation, data curation, formal analysis. **Myungsuk Kim:** conceptualization, data curation, formal analysis, writing – original draft. **Tam Thi Le:** investigation, data curation. **Geon Park:** investigation, data curation. **Yuna Jung:** investigation, data curation. **Hyobin Jeong:** data curation. **Youngsang Yoo:** supervision, project administration, funding acquisition. **Wook‐Bin Lee:** conceptualization, supervision, project administration, writing – original draft. **Sang Hoon Jung:** supervision, project administration, funding acquisition.

## Funding

This work was supported by the Korea Institute of Science and Technology, 26Z9061 and Gangneung Asan Hospital, 2024II0004.

## Conflicts of Interest

The authors declare no conflicts of interest.

## Supporting information


**Figure S1:** Schematic diagram of extraction and fractionation of *C. nomame* extract into organic solvent fractions (upper).

**Figure S2:** Cytotoxicity and β‐hexosaminidase activity of CN on RBL‐2H3 cells.

**Figure S3:** Experimental design of the in vivo study using CN.

**Figure S4:** Changes in OVA‐stimulated pulmonary immune cells following CN treatment.

**Figure S5:** HPLC chromatogram and structures of isolated compounds from CN.

**Figure S6:** Differentiation of bone marrow‐derived mast cells (BMMCs) over 28 days.

**Figure S7:** Anti‐allergic effects of luteolin isolated from CN in bone marrow‐derived mast cells.

**Figure S8:** Effects of luteolin on cell viability in BMMCs and THP‐1 cells.

**Table S1:** HPLC conditions and identified compounds isolated from CN.

**Table S2:** Primers for real‐time quantitative PCR.

**Appendix S1:** ptr70363‐sup‐0011‐AppendixS1.docx.

## Data Availability

The data that support the findings of this study are available in paper or the Supporting Information of this article. The raw and processed sequencing data reported in this paper are available from Gene Expression Omnibus (GEO): GSE281090.
