# Supplementary figures and images for: Anti‐Allergic Potential of Chamaecrista nomame and Its Compound Luteolin for Novel Asthma Therapy
Source: Phytother Res. 2026 May 5;40(7):4309–20. doi: 10.1002/ptr.70363 (PMC13340961; doi:10.1002/ptr.70363)

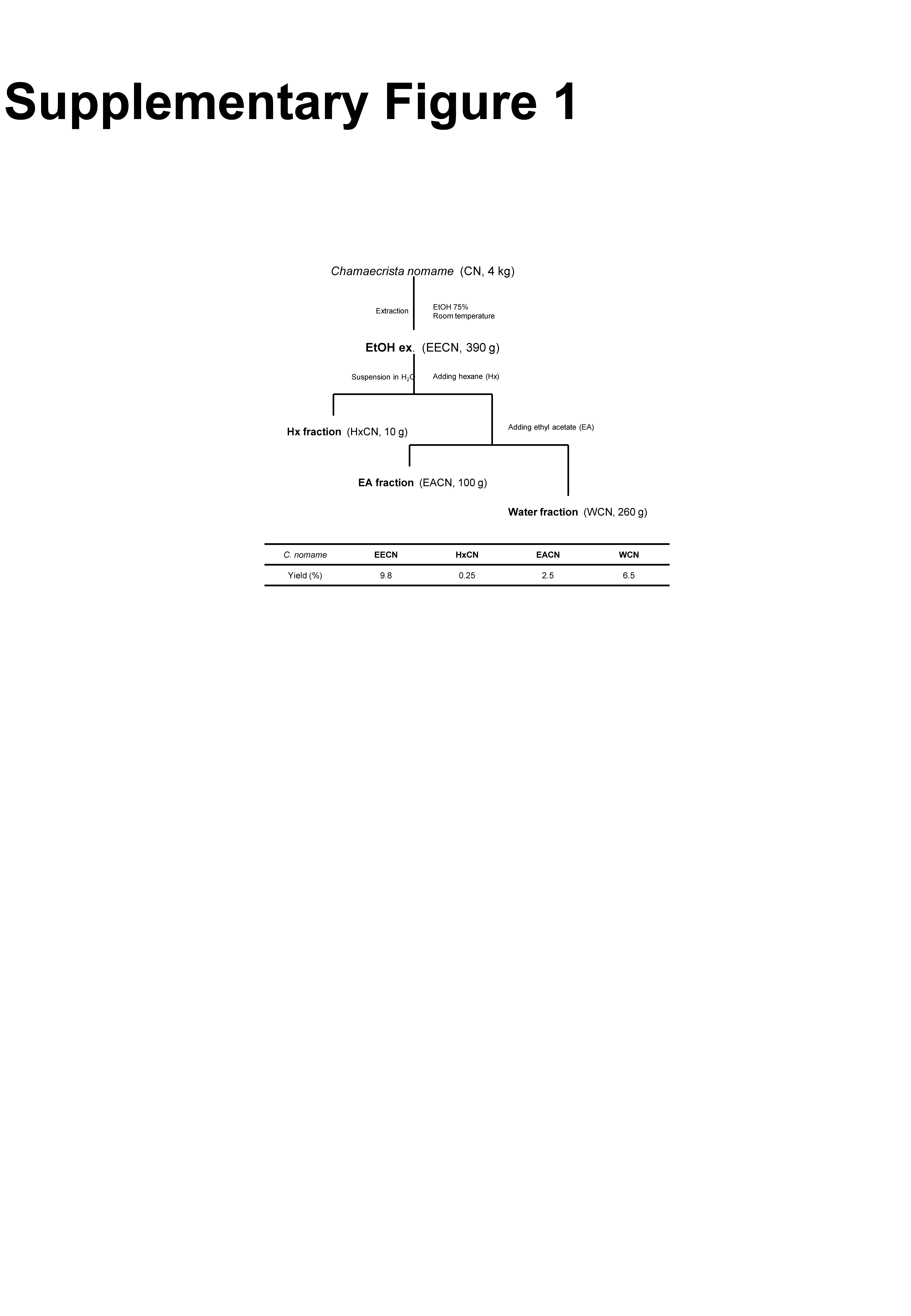

Supplement: Supplementary file 1 — Figure S1: Schematic diagram of extraction and fractionation of C. nomame extract into organic solvent fractions (upper). [file PTR-40-4309-s006.tif]

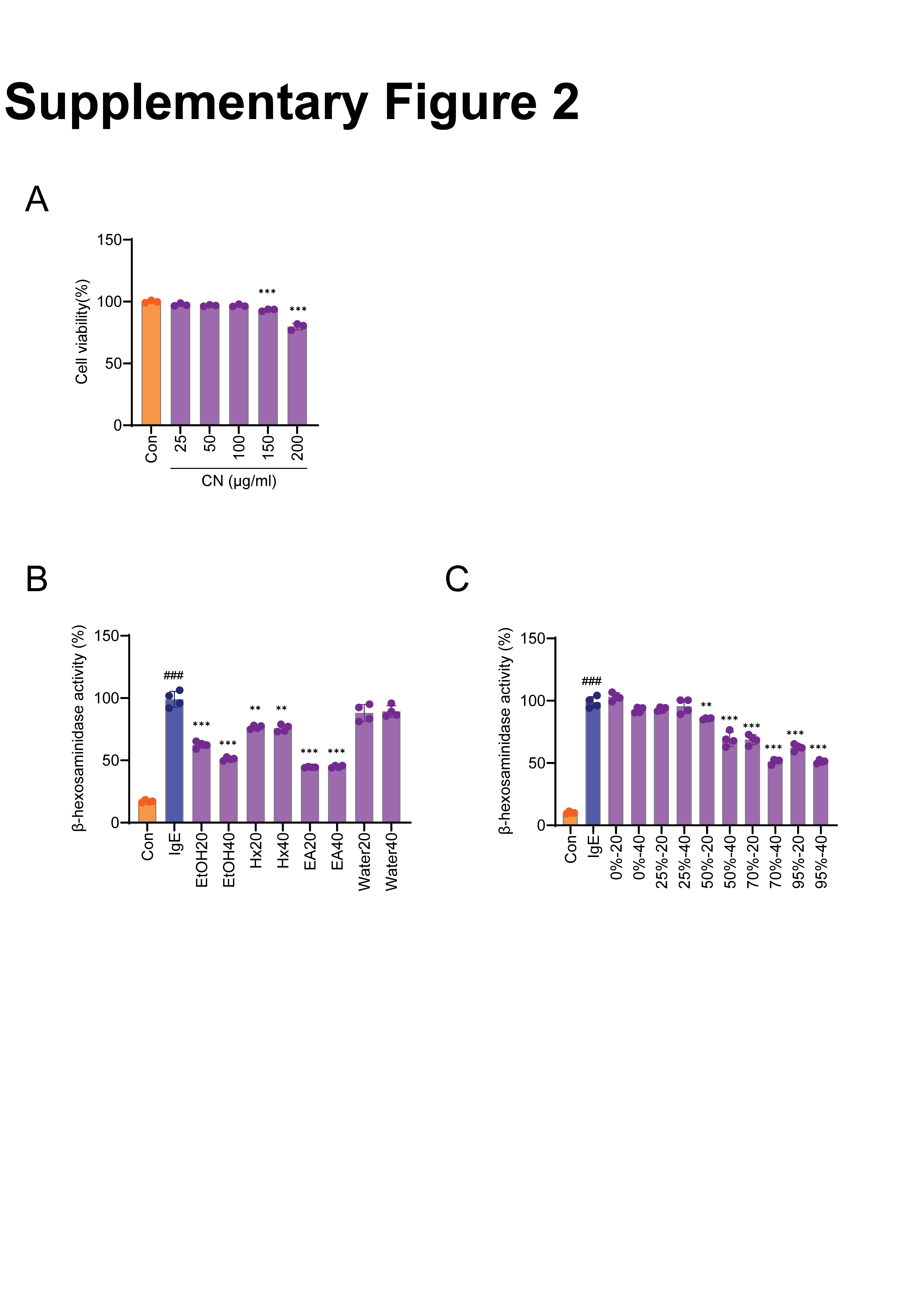

Supplement: Supplementary file 2 — Figure S2: Cytotoxicity and β‐hexosaminidase activity of CN on RBL‐2H3 cells. [file PTR-40-4309-s008.tif]

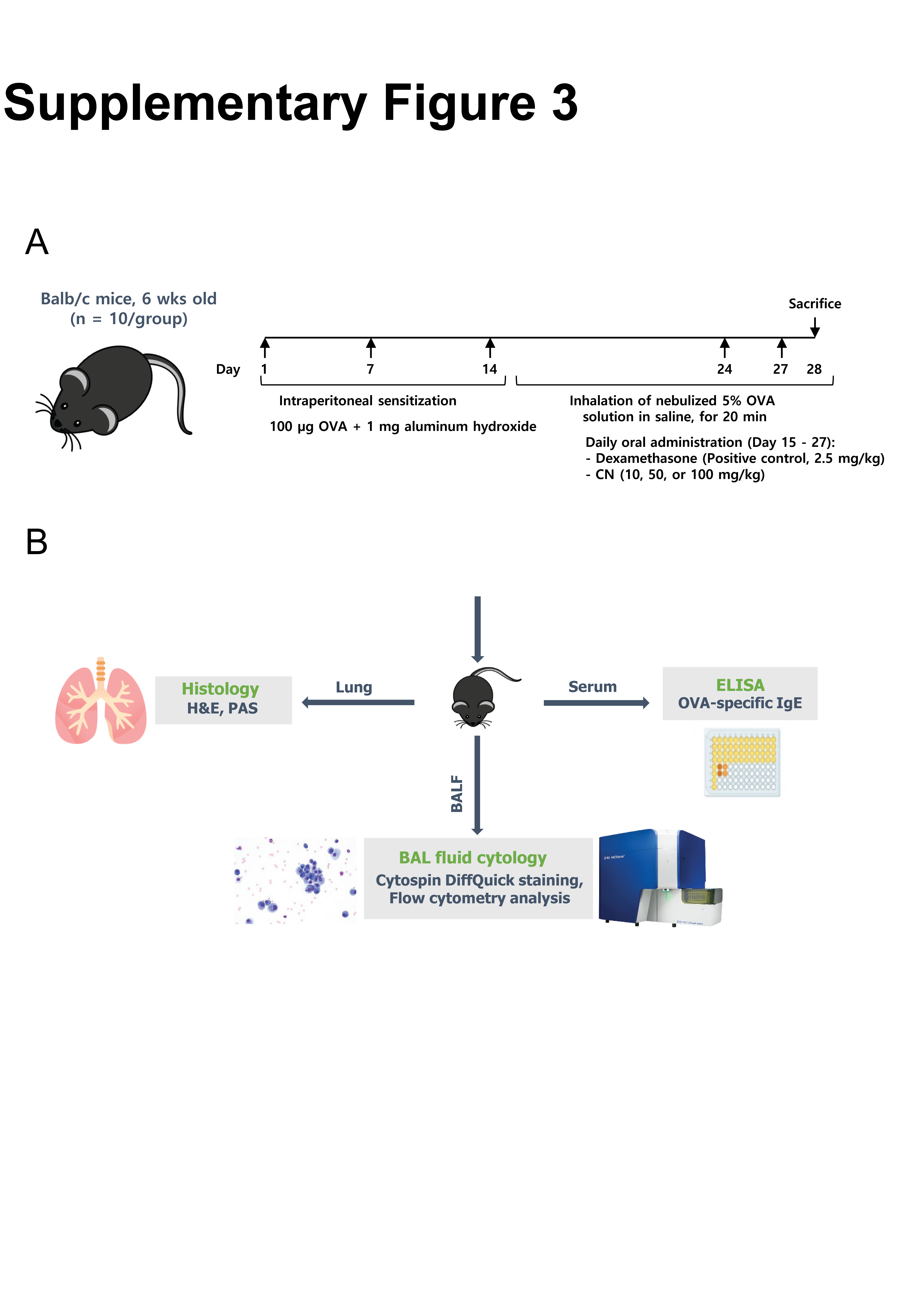

Supplement: Supplementary file 3 — Figure S3: Experimental design of the in vivo study using CN. [file PTR-40-4309-s010.tif]

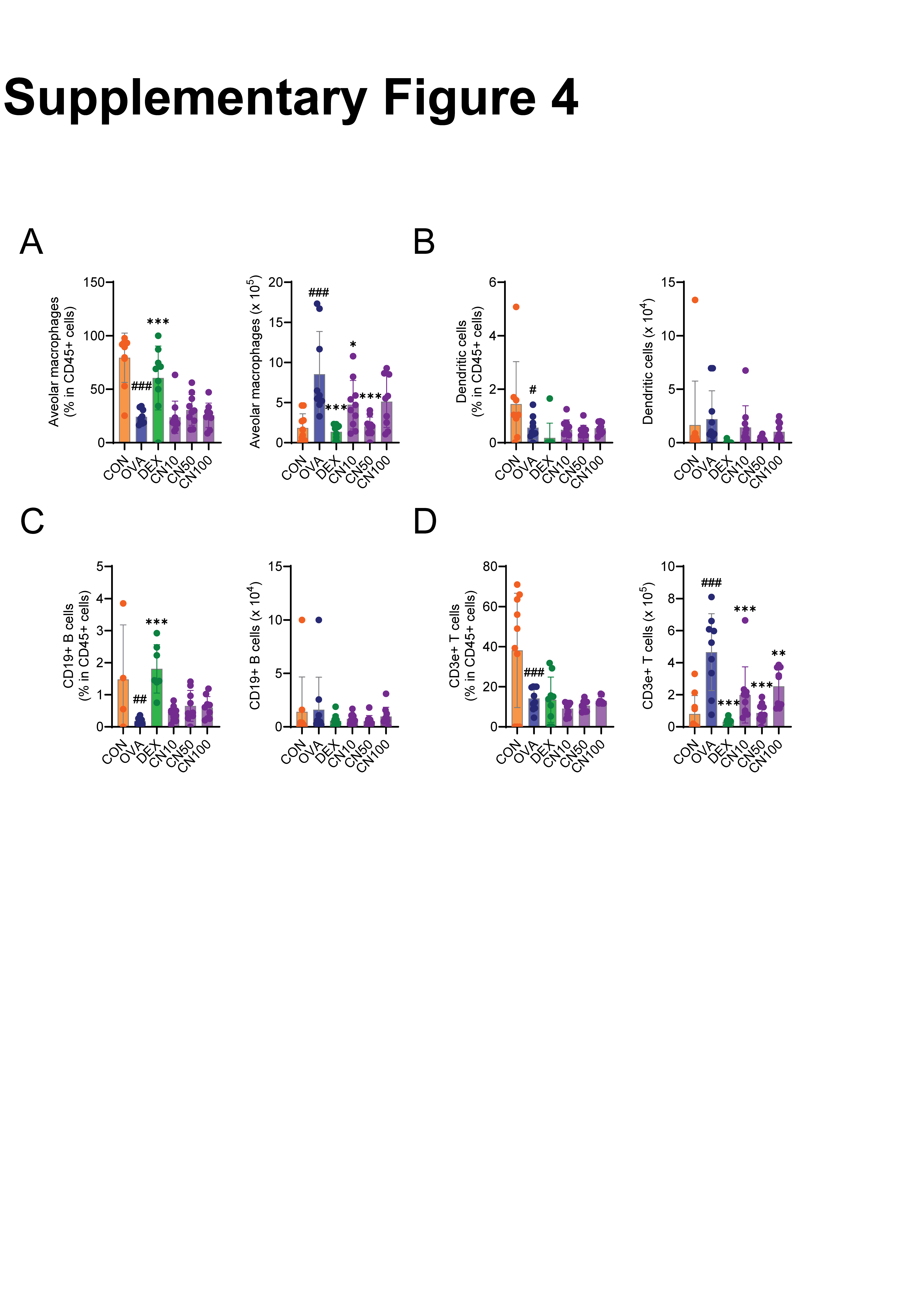

Supplement: Supplementary file 4 — Figure S4: Changes in OVA‐stimulated pulmonary immune cells following CN treatment. [file PTR-40-4309-s004.tif]

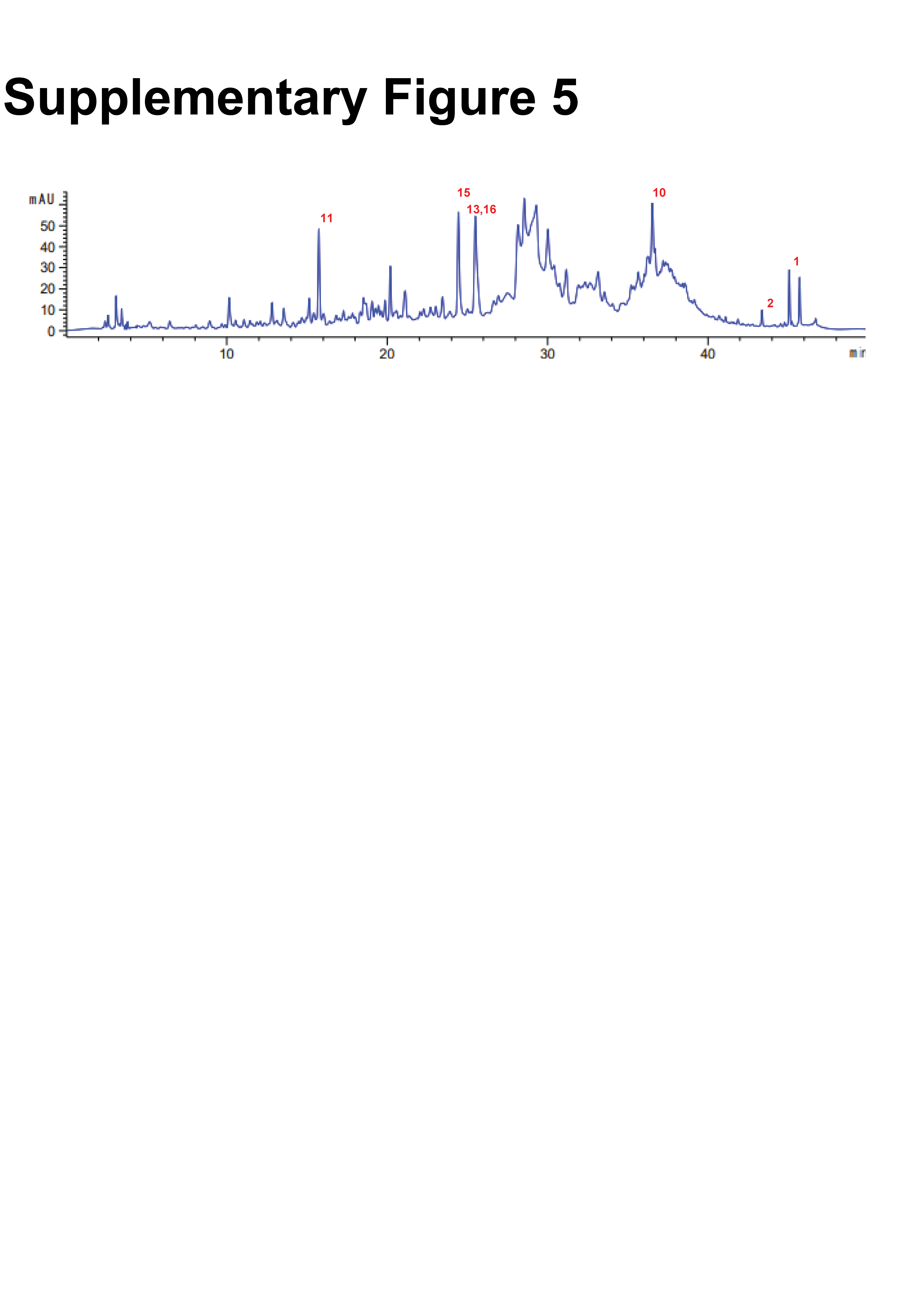

Supplement: Supplementary file 5 — Figure S5: HPLC chromatogram and structures of isolated compounds from CN. [file PTR-40-4309-s011.tif]

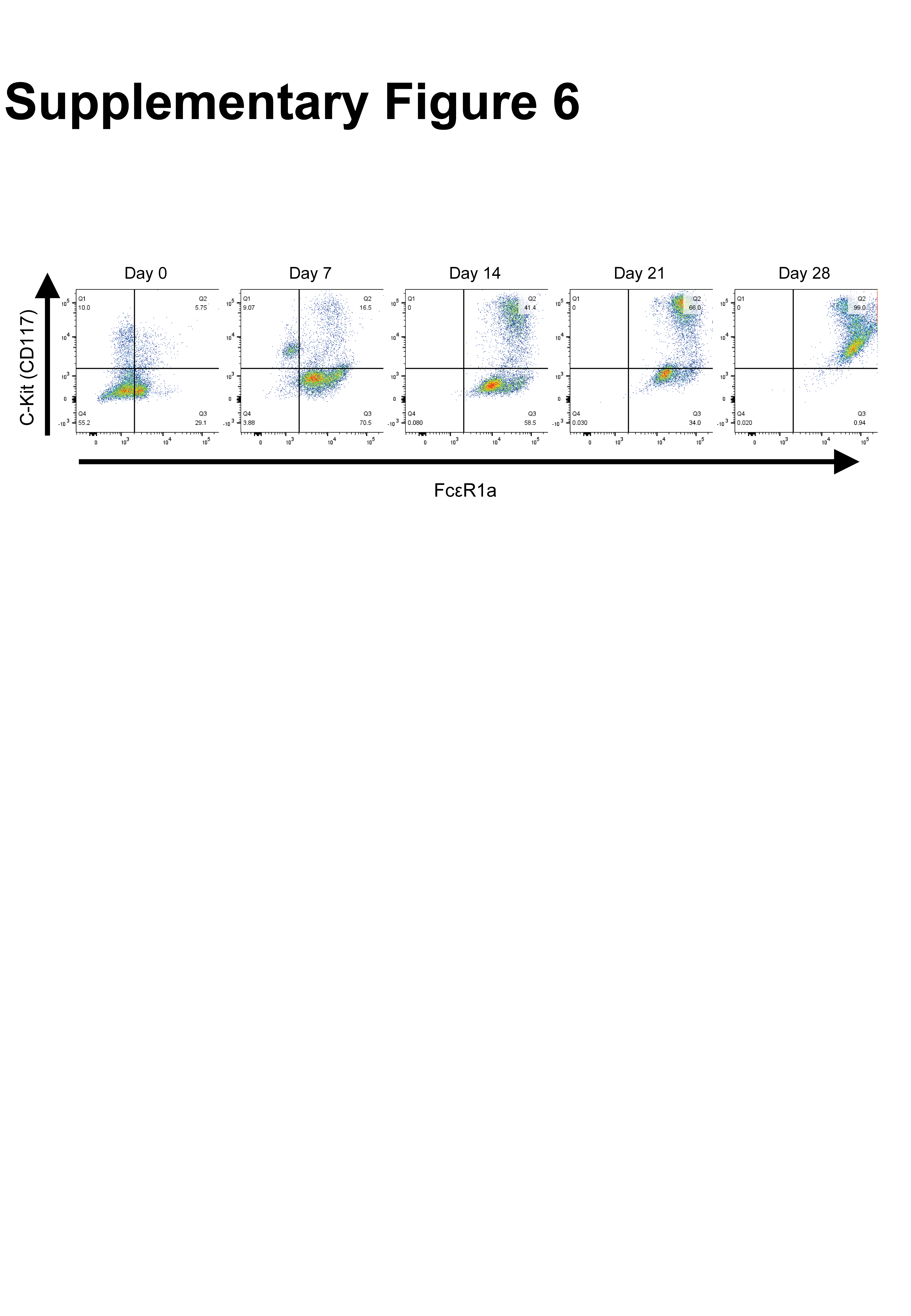

Supplement: Supplementary file 6 — Figure S6: Differentiation of bone marrow‐derived mast cells (BMMCs) over 28 days. [file PTR-40-4309-s009.tif]

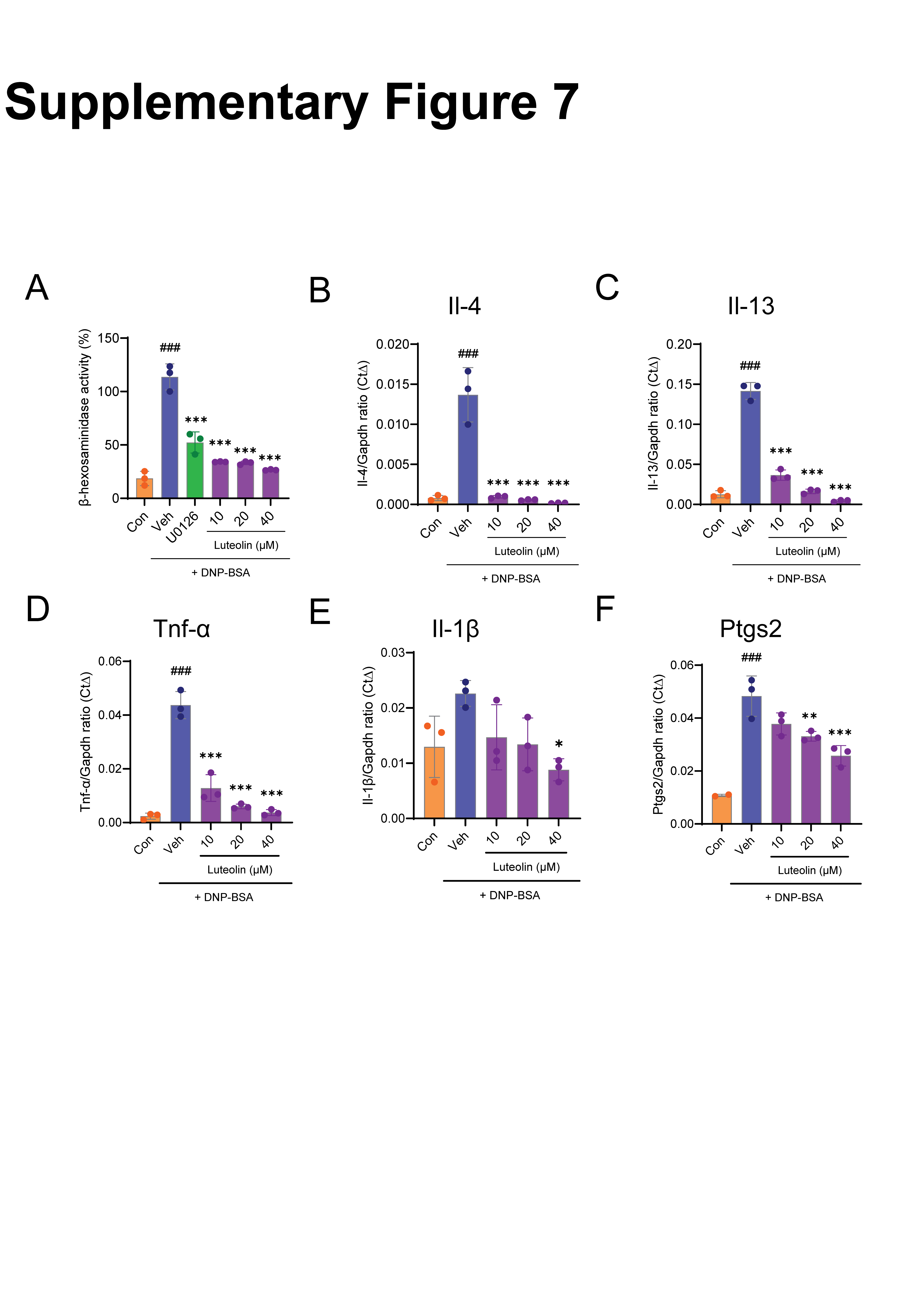

Supplement: Supplementary file 7 — Figure S7: Anti‐allergic effects of luteolin isolated from CN in bone marrow‐derived mast cells. [file PTR-40-4309-s005.tif]

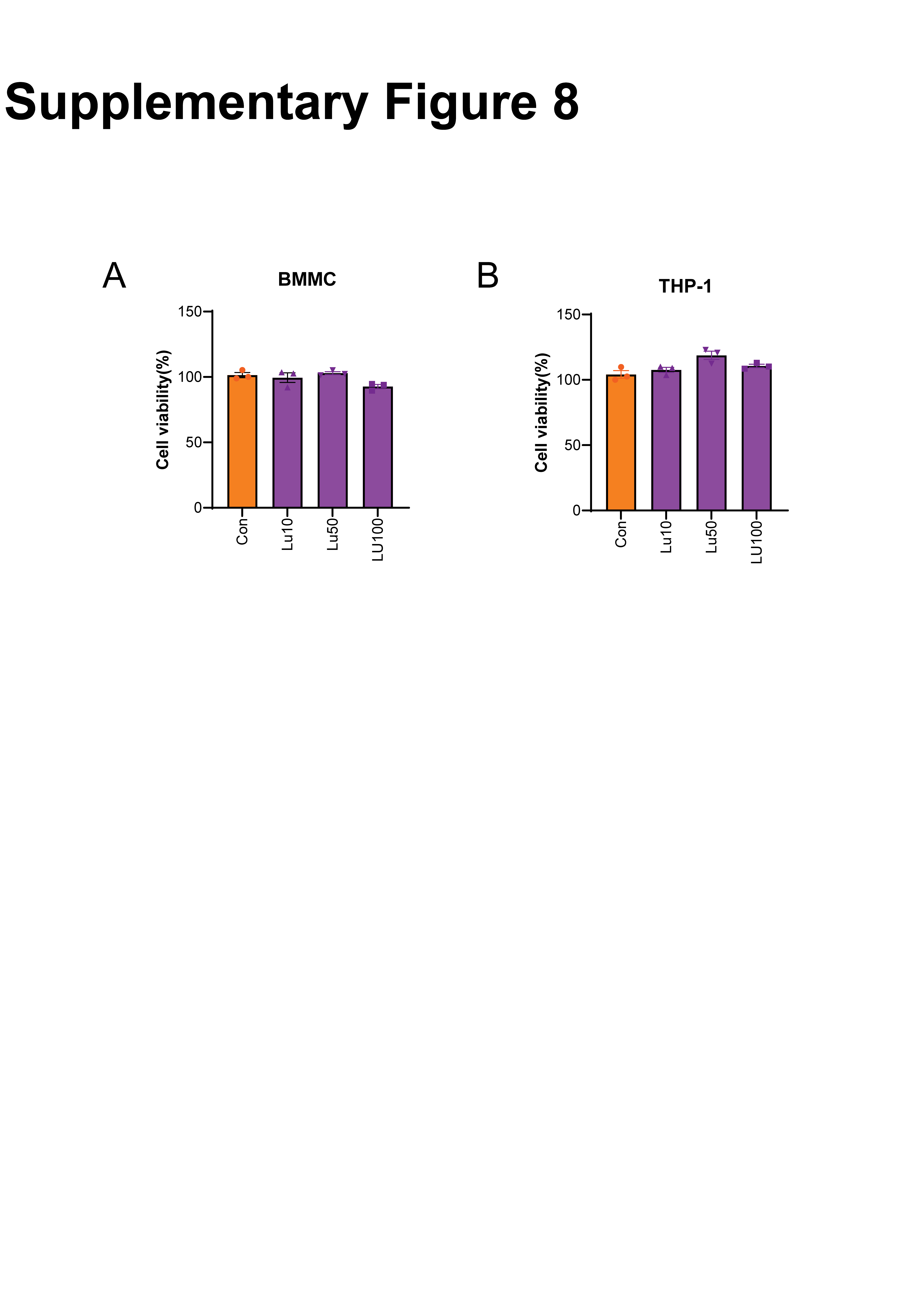

Supplement: Supplementary file 8 — Figure S8: Effects of luteolin on cell viability in BMMCs and THP‐1 cells. [file PTR-40-4309-s001.tif]
