# Supplementary material for: Anti‐Allergic Potential of Chamaecrista nomame and Its Compound Luteolin for Novel Asthma Therapy
Source: Phytother Res. 2026 May 5;40(7):4309–20. doi: 10.1002/ptr.70363 (PMC13340961; doi:10.1002/ptr.70363)
Supplement: Supplementary file 11 — Appendix S1: ptr70363‐sup‐0011‐AppendixS1.docx. [file PTR-40-4309-s003.docx]

**Anti-Allergic Potential of *Chamaecrista nomame* and its Compound Luteolin for Novel Asthma Therapy.**

**Appendix S1: Supplementary Materials and Methods**

*1.1. High-performance liquid chromatography (HPLC) profiling of CN*

HPLC analysis was conducted using an Agilent Series 1200 liquid chromatography system equipped with a YMC Hydrosphere C18 column (4.6 × 250 mm, 5 µm particle size), maintained at 30°C. The HPLC protocol is detailed in **Supplementary Table 1**. To enhance peak resolution, 0.1% formic acid was added to both the acetonitrile and water mobile phases. The injection volume was set at 10 µL, with a flow rate of 1.0 mL/min. UV detection was performed at 280 nm. For sample preparation, 10 mg of the CN was dissolved in 1 mL of methanol, and an aliquot of this solution was subjected to HPLC analysis.

*1.2. Compounds Isolation and Identification of CN*

The EA-soluble fraction was subjected to silica gel column chromatography, employing a stepwise gradient of Hx-EA (15:1 to 0:15), to yield 34 subfractions (CMEA 1–34). Subfractions CMEA 1, 6, 10, 23, 26, and 32 were further purified using a Sephadex LH-20 column, followed by preparative medium-pressure liquid chromatography, to yield the following compounds: **1** (1.9 mg) from CMEA-1; **2** (2.8 mg) from CMEA-6; **3** (1.3 mg), **4** (2.7 mg), **5** (4.6 mg), **6** (2.6 mg), **7**/**8** mixture (2.6 mg), **9** (2.5 mg), and **12** (4.8 mg) from CMEA-10; **10** (4.4 mg) from CMEA-23; **11** (5.6 mg) from CMEA-26; and **13** (5.8 mg), **14** (2.6 mg), **15** (3.5 mg), **16** (3.1 mg), and **17** (1.9 mg) from CMEA-32. *Luteolin (****10****)*: yellowish powder; ^1^H NMR (500 MHz, DMSO-*d*_6_) *δ* 7.43 (dd, *J* = 8.3, 2.3 Hz, 1H), 7.40 (d, *J* = 2.2 Hz, 1H), 6.90 (d, *J* = 8.3 Hz, 1H), 6.68 (s, 1H), 6.45 (d, *J* = 2.1 Hz, 1H), 6.20 (d, *J* = 2.0 Hz, 1H). ^13^C NMR (125 MHz, DMSO-*d*_6_) *δ* 182.13, 164.58, 164.36, 161.95, 157.75, 150.16, 146.20, 121.97, 119.46, 116.48, 113.84, 104.17, 103.34, 99.29, 94.30.

*1.3. Chemicals and Apparatus*

NMR spectra, including both 1D and 2D analyses, were recorded on a Bruker Avance DRX Spectrometer, with chemical shifts reported in *δ* (ppm). Open column chromatography was performed using silica gel (63–200 µm). For thin-layer chromatography (TLC), Merck silica gel 60 F254 plates and RP-18 F254 plates were employed. Isolated compounds were visualized by spraying with 20% H_2_SO_4_ and heating for 5 minutes. Analytical-grade acetonitrile and HPLC-grade water were sourced from Fisher Scientific (Pittsburgh, PA, USA). Silica gel was obtained from Merck (Darmstadt, Germany), and Sephadex LH-20 was purchased from Pharmacia (Uppsala, Sweden) for use in open column chromatography.

*1.4. Chemicals and Reagents*

Dexamethasone (Dex), Aluminum hydroxide (Al(OH)₃) and ovalbumin (OVA) was purchased from Sigma Chemical Co. (MO, USA). Phosphate-buffered saline (PBS), Dulbecco's Modified Eagle Medium (DMEM) was purchased from Corning (NY, USA). Fetal bovine serum (FBS) was purchased from Gibco (MA, USA). Ficoll-Paque PLUS was purchased from GE Healthcare (IL, USA), PAS stain kit ab150680, IL-4 and TNF-α ELISA kits was purchased from Abcam (CB, UK). Diff-Quik stain kit was purchased from Siemens (DE, USA). LEGEND MAX™ Human B cell panel, LEGEND MAX™ Mouse B cell panel, Fluorescein isothiocyanate (FITC)-conjugated anti-CD11c, Anti-CD11b, Phycoerythrin (PE)-conjugated anti-Ly6G, Anti-CD170, and anti-F4/80 was purchased from BioLegend (CA, USA). Anti-mouse CD16/CD32 was purchased from BD Biosciences (CA, USA). EZ-cytox kit purchased from DOGEN (Seoul, Korea).

*1.5. Experimental Animals*

Female BALB/c mice, six weeks old, were obtained from Orient Bio Inc. (Seong-nam, Korea). The mice were housed in an environmentally controlled facility under a 12-hour light/dark cycle, at a constant temperature of 23 ± 2°C, and relative humidity maintained at 55 ± 10%. All experimental procedures involving animals were conducted in accordance with the ethical guidelines and standards approved by the KIST Animal Care and Use Committee (approval NO. KIST-5088-2022-03-031).

*1.6. Induction of Asthma*

A total of 60 female mice were randomly divided into six groups, each consisting of 10 mice. The groups were designated as follows: control group, OVA group, OVA + DEX group (positive control with dexamethasone), OVA + CN 10 group, OVA + CN 50 group, and OVA + CN 100 group. Dexamethasone served as the positive control drug. The asthma model was induced using a previously established protocol. Briefly, each mouse was sensitized intraperitoneally with 100 μg of OVA and 1 mg of Al(OH)₃ in 200 μL of PBS on days 0, 7, and 14. On days 24 and 27, OVA-challenged mice were exposed to ultrasonic atomization of 5% OVA for 20 minutes each day. The control group received sensitization and challenges with an equivalent volume of PBS instead of OVA. From day 15 to day 27, mice in each group received daily oral administration of varying doses of CN (10 mg/kg, 50 mg/kg, and 100 mg/kg), DEX (2.5 mg/kg), or an equivalent volume of 2% sodium carboxymethylcellulose solution. Mice were sacrificed 24 hours after the final challenge, and relevant samples were collected for subsequent analyses.

*1.7. Histological Assessment of Asthma*

Lung tissues from the experimental groups were fixed in 4% paraformaldehyde, followed by dehydration, paraffin embedding, and sectioning. Sections of lung tissue were then subjected to H&E and PAS for histological assessment. For H&E staining, the slides were treated with hematoxylin buffer at room temperature, rinsed three times with distilled water, and immersed in a 1% eosin Y solution. PAS staining was performed using a PAS stain kit (ab150680; Abcam, United Kingdom) according to the manufacturer's instructions. Toluidine blue staining was performed following a conventional protocol. The slides were treated with toluidine blue working solution for 3min. Stained slides were mounted and examined under an EVOS XL Core Imaging system microscope (Invitrogen, Life Technologies, California, USA).

*1.8. Collection of Bronchoalveolar Lavage Fluid (BALF) and Serum, and Leukocyte Counts*

Twenty-four hours after the final challenge, the trachea was intubated, and the lungs were lavaged twice with 0.5 mL of PBS to obtain BALF. The collected BALF was centrifuged at 1500 × g for 10 minutes, and the supernatants were carefully harvested and stored at –80°C for subsequent cytokine detection. The cell pellet from the BALF was resuspended in 100 µL of PBS, and differential leukocyte counts, including PMNs, lymphocytes, and monocytes, were performed on Cytospin slides stained with Diff-Quik (Siemens, Newark, DE, USA). For serum collection, blood was drawn from the abdominal caval vein after anesthesia and an abdominal incision. The collected blood samples were centrifuged at 300 × g for 10 minutes at 4°C, and serum samples (at least 200 μL) were collected and stored at –80°C for subsequent analyses.

*1.9. Measurement of Cytokines and IgE*

Serum cytokine levels were quantified using a 13-plex cytometric bead array (LEGENDplex™ Mouse B cell Panel, BioLegend), following the manufacturer’s instructions. This kit allows for the simultaneous measurement of 13 cytokines: IL-4, IL-6, IL-12p70, IL-17A, IL-2, TNF-α, TGF-β1, IL-13, IFN-γ, BAFF, BCMA, sCD40L, and IL-10. Serum levels of OVA-specific IgE were determined using an enzyme-linked immunosorbent assay (ELISA) kit (LEGEND MAX™ Mouse OVA-specific IgE ELISA Kit, BioLegend), as per the manufacturer's protocol.

*1.10. Flow Cytometry*

Single-cell suspensions were prepared from BALF. To block nonspecific Fc receptor binding, cells were incubated with anti-mouse CD16/CD32 antibody (BD Biosciences). The blocked cell suspensions were subsequently stained with a panel of monoclonal antibodies for flow cytometry analysis. The antibodies used included allophycocyanin (APC)-conjugated anti-CD45 (BioLegend), fluorescein isothiocyanate (FITC)-conjugated anti-CD11c and anti-CD11b (BioLegend), and phycoerythrin (PE)-conjugated anti-Ly6G, anti-CD170, and anti-F4/80 (BioLegend). Stained cells were analyzed using a BDVerse flow cytometer, with data acquisition performed using FACSSuite software (BD Biosciences). Data analysis was conducted using FlowJo software (Treestar, Ashland, OR, USA).

*1.11. Cell culture*

The RBL-2H3 cell line was obtained from the Korean Cell Line Bank, Korean Cell Line Research Foundation (Seoul, Korea). Cells were cultured in Dulbecco's Modified Eagle Medium (DMEM) supplemented with 10% fetal bovine serum (FBS) and 100 U/mL penicillin/streptomycin. Cultures were maintained in a controlled environment at 37°C with 5% CO_2_.

*1.12. Bone marrow-derived mast cells (BMMCs)*

Bone marrow cells were isolated from C57BL/6 mice and subsequently cultured in DMEM that was enriched with 30% WEHI-3-conditioned medium and 20% heat-inactivated FBS. To support cell growth and prevent contamination, the medium also contained 100 U/mL of penicillin and streptomycin. The medium was replaced with fresh solution twice a week to maintain optimal growth conditions. The cell cultures were maintained in a controlled environment at a temperature of 37°C and a CO2 concentration of 5%. This setup was sustained for at four weeks to facilitate the maturation and achieve a high purity of BMMCs.

*1.13. Peripheral blood mononuclear cells (PBMCs)*

Before commencement, the study protocol was thoroughly reviewed and authorized by the Institutional Review Board at Asan Medical Center (IRB approval number: 2024-09-007). Participants were selected from a hospital-maintained database and all participants provided written informed consent. Peripheral blood mononuclear cells were isolated from the peripheral blood of human donors (age range: 19-79 years) following informed consent. Blood was collected into heparinized vacutainer tubes and processed within 2 hours of collection. Whole blood was first diluted 1:1 with PBS and carefully layered onto 1 volume of Ficoll in a 50 ml conical tube. The sample was then centrifuged at 400 x g for 30 min at room temperature with no brake. After centrifugation, the PBMC layer was located at the interface between the plasma and Ficoll. The mononuclear cells were carefully aspirated and transferred to a fresh tube, followed by washes with PBS to remove residual Ficoll and plasma. For culture, PBMCs were seeded at 1 x 10^6^ in 96 well culture plate and incubated at 37°C with 5% CO_2_.

*1.14. Cell Viability Assay*

To assess the impact of CN or its isolated compounds on the viability of RBL-2H3 cells or BMMCs, the EZ-Cytox kit (DOGEN, Seoul, Korea) was used. Briefly, cells were seeded at a density of 1 × 10^4^ cells per well and treated with varying concentrations of CN or compounds for 24 hours. The EZ-Cytox reagent was then added, and the cells were incubated at 37°C for 30 minutes. Absorbance was measured at 450 nm using a microplate reader to determine cell viability.

*1.15. ß-Hexosaminidase Assay*

RBL-2H3 or BMMC cells were seeded at a density of 5 × 10^5^ cells per well in a 24-well plate and sensitized with DNP-IgE (50 ng/mL) for 24 hours. Following a 1-hour pre-treatment with CN or isolated compounds, the cells were stimulated with DNP-BSA (100 ng/mL) in Siraganian buffer (119 mM NaCl, 5.6 mM glucose, 0.4 mM MgCl2, 0.1% BSA, 5 mM KCl, 25 mM PIPES, 1 mM CaCl2, pH 7.2). The β-hexosaminidase enzymatic activity was assessed by measuring the color change of the substrate, 1 mM p-nitrophenyl-N-acetyl-d-glucosaminide, in citrate buffer. Absorbance was measured at 450 nm using a microplate reader. Histamine release in the collected supernatants was quantified using a histamine enzyme immunoassay (EIA) kit (Oxford Biomedical Research, Rochester Hills, MI, USA).

*1.16. Inflammatory Cytokine and Prostaglandin E2 Release*

RBL-2H3 cells were pre-treated with varying concentrations of CN or its isolated compounds for 1 hour before being sensitized overnight with DNP-IgE (50 ng/mL). Following stimulation with DNP-BSA (100 ng/mL) for 24 hours, supernatants were collected for analysis of cytokines and prostaglandin E2 (PGE2). IL-4 and TNF-α concentrations were measured using ELISA kits (Abcam, UK), and PGE2 levels were quantified using an EIA kit (Cayman Chemical, Ann Arbor, MI, USA).

*1.17. PBMCs stimulation with HDM and cytokine measurement*

PBMCs were stimulated with House Dust Mite (HDM) extract at a final concentration of 2 μg for 24 hours. After the stimulation period, the cultured supernatants were collected, clarified by centrifuged at 1,500 x g for 10 minutes at 4°C and stored at -80°C until cytokine analysis. Cytokine levels in the supernatants were quantified using the LEGEND MAX™ Human B cell panel. The assay was performed following the provided protocol.

*1.18. Real-time Quantitative PCR*

Total RNA was extracted from BMMCs using the RNeasy Mini Kit (QIAGEN, Germany). cDNA synthesis was performed using SuperScript III Reverse Transcriptase (Invitrogen, USA). Subsequently, quantitative PCR was conducted using the TaqMan Gene Expression Assay with TaqMan Fast Advanced Master Mix and a QuantStudio 6 System (Thermo Fisher Scientific, USA). Data were analyzed using the 2−ΔΔCt method according to the manufacturer's instructions. The threshold cycle (Ct) values were normalized to glyceraldehyde-3-phosphate dehydrogenase and analyzed using the 2−ΔΔCt method to calculate relative fold changes in gene expression. Primer sequences are provided in **Supplementary Table 2**.

*1.19. RNA Sequencing and Data Analysis*

Total RNA was extracted using the HybridR RNA Isolation Kit (GeneAll, Seoul, Korea), following the manufacturer's protocol. RNA quality and quantity were assessed using the 2100 Bioanalyzer System (Agilent Technologies, Amstelveen, Netherlands) and the NanoDrop™ 2000 Spectrophotometer (Thermo Fisher). RNA libraries were prepared using the TruSeq Stranded mRNA Library Prep Kit (Illumina, CA, USA) and sequenced on a NovaSeq 6000 instrument (Illumina, CA, USA). Raw reads were filtered to remove contaminants, adaptor sequences, and low-quality reads using HTStream (v1.1.0). The processed data were aligned to the reference genome using STAR (version 2.7.0f). Custom R scripts were used to assess read and alignment quality, compile counts, and filter transcripts with a median CPM > 1 across samples (15,252 transcripts; GSE281090).

*1.20. Differential Gene Expression and Enrichment Analysis*

Differentially expressed genes (DEGs) were identified using the "limma" package (v3.11) in R on TMM-normalized log2-transformed CPM values. Enrichment analysis of DEGs was performed using enrichR to identify significantly enriched terms and pathways in Gene Ontology (GO) Biological Process 2023 and Kyoto Encyclopedia of Genes and Genomes (KEGG) 2019 Mouse. This analysis highlights functional categories specific to the upregulated and downregulated genes within the dataset. Notably, GO Biological Process 2023 includes 5,103 terms and 14,433 genes, with individual terms categorized within broader functional classes but not strictly confined to a hierarchical structure. Each identified GO term has a unique annotation number linked to a specific function. Both the Gene Ontology website and enrichR utilize the full set of available terms, not relying on a specific hierarchy, for comprehensive enrichment analysis.

*1.21. Statistical analysis*

Statistical analysis was performed using R software (version 4.2.2) and GraphPad Prism software (version 10.0; GraphPad, San Diego, CA, USA). A two-tailed *t*-test with a 95% confidence interval was used to calculate P values. The figure legends provide details on group sizes, repeatability, and P values for each experiment.
